# Preoperative Plasmapheresis for Elective Thymectomy in Myasthenia Patient: Is It Necessary?

**DOI:** 10.1155/2013/238783

**Published:** 2013-06-11

**Authors:** Somcharoen Saeteng, Apichat Tantraworasin, Sophon Siwachat, Nirush Lertprasertsuke, Juntima Euathrongchit, Yuttaphan Wannasopha

**Affiliations:** ^1^General Thoracic Unit, Department of Surgery, Faculty of Medicine, Chiang Mai University Hospital, Chaing Mai 50200, Thailand; ^2^Department of Pathology, Faculty of Medicine, Chiang Mai University Hospital, Chaing Mai 50200, Thailand; ^3^Department of Radiology, Faculty of Medicine, Chiang Mai University Hospital, Chaing Mai 50200, Thailand

## Abstract

*Background*. Role of plasmapheresis before thymectomy remains controversial. The aim of this study is to determine the peri-operative and post-operative outcome of a thymectomy between performing and not performing a pre-operative plasmaphreresis. *Patients and Methods.* A retrospective chart review study was conducted in Chiang Mai University Hospital between January 2006 and December 2011. There were 86 myasthenia patients divided into two groups; Preoperative plasmapheresis group (PPG) and no preoperative plasmapheresis group (NPPG). The primary outcome involved post-operative extubation and the secondary outcome included post-operative complications, 28 day mortality and length of hospital stay. *Results.* Eighty-six patients were enrolled in this study. The number of patients who had a history of myasthenic crisis at any time or within one month in the PPG was significantly more than those in the NPPG. Muscle power and forced expiratory vital capacity in the NPPG was higher than that in the PPG. The postoperative extubation rate was similar in both groups. After controlling for the propensity score, there were no statistically significant differences in both of primary and secondary outcomes. *Conclusion*. The results of this study shows no significant differences between both groups in all outcomes, therefore the pre-operative plasmaphresis is not necessary for elective thymectomy.

## 1. Introduction 

Myasthenia gravis is an acquired, neuromuscular, autoimmune disorder. The basic pathogenesis of the disease understood so far is the production of autoantibodies against the acetylcholine receptors of the endplate [1, 2] and thereby causing immunologic destruction and reduction of the number of the receptors. The miniature endplate potential amplitude is decreased, and the endplate potentials are largely subthreshold leading to easy fatigability and weakness. The thymus is believed to play an integral role in the pathogenesis of myasthenia gravis. The role of the thymus in the development of antibodies against the acetylcholine receptors has been clearly established; therefore the relationship between myasthenia and thymic abnormalities has been suggested [2, 3]. The myasthenia patient presents with a varied degree of muscle weakness such as ptosis (ocular involvement), proximal muscle weakness, respiratory failure (involving respiratory muscle), or dysphagia (bulbar involvement).

Current treatment strategies include anticholinesterases for the minimal symptoms patients while nonresponders require treatment with steroids and immunosuppressants. Considering the role of the thymus in the pathogenesis, complete removal of the thymus has become a standard procedure for the management of myasthenia gravis with remarkable and sustained improvement in many. Although the complete remission rate has progressively increased from 37.4% to 58.2% and 75% at three years, ten years, and 15 years of followup, respectively [4], the myasthenia crisis is still a life-threatening major complication of thymectomy from respiratory failure requiring intubation and mechanical ventilation [3].

In 1977, Pinching et al. [5] advocated plasmapheresis by decreasing the circulating antibodies. Plasmapheresis reduces the antiAch receptor antibody titer in myasthenia gravis and alleviates symptoms. However its effects are short lasting as it cannot prevent resynthesis, and there are several adverse effects of plasmapheresis including hypotension, coagulopathy, catheter-related complications, and high cost that has brought plasmapheresis into question as to whether it should be routinely used or only in selected cases.

In Thailand the cost of plasmapheresis is high, approximately 20,000–40,000 Thai Baht (680–1400 US Dollar). This cost is not included in our government health insurance. Patients who are scheduled for a preoperative plasmapheresis must pay this cost by themselves. Although some patients who could not afford the cost refused to have a preoperative plasmapheresis, the thymectomy was still performed. Low post-operative complications occurred, and the endotracheal tube was able to be immediately removed after the operation. The main concern in this situation is whether a preoperative plasmapheresis is necessary or not.

The aim of this study was to evaluate the necessity of preoperative plasmapheresis for patients who were treated with an elective thymectomy. My hypothesis is that a preoperative plasmapheresis is not necessary in myasthenic patients who undergo an elective thymectomy because the outcomes of both treatments are the same.

## 2. Material and Methods

 This study was a retrospective cohort efficacy research study. A total of 86 patients, 25 males and 61 females aged 15–69 years, with a diagnosis of MG based on clinical and pharmacological criteria that underwent a thymectomy from January 1 2005, to December 31 2011, in Chiang Mai University Hospital were enrolled in this study. Ethical approval was obtained from the joint institutional research ethics committees of the Chiang Mai University. All patients received a preoperative evaluation by a neurologist. The decision to perform a preoperative plasmapheresis was done by a neurologist. Indications for preoperative plasmapheresis in our institutes included receiving a high dose steroid (more than one mg/kg/day), history of myasthenic crisis or respiratory failure at any time, or low vital capacity before surgery. There are no guidelines or consensus regarding preoperative plasmapheresis in MG patients scheduled for elective thymectomy. Patients who received and could support the cost of preoperative plasmapheresis were allocated to the preoperative plasmapheresis group (PPG), and patients who did not received or could not support the cost of it were allocated to the no preoperative plasmapheresis group (NPPG). The preoperative evaluation included forced vital capacity (FVC), history of myasthenic crisis, and grading of muscle weakness before surgery to evaluate the necessity for performing preoperative plasmapheresis. All cases had good motor power and neither respiratory failure nor bulbar involvement before undergoing an elective thymectomy. In the PPG group plasmapheresis was performed once in the surgical intensive care unit one day prior to thymectomy.

 The computerized database and the medical records of these patients treated with selective thymectomy were reviewed. The severity of the MG (severity before operation) was evaluated according to Osserman and Genkins [6] as follows: I, ocular disease only; IIA, mild generalized with no prominent bulbar signs; IIB, moderate generalized, no crisis; III, acute fulminating generalized signs with prominent bulbar involvement and crisis; and IV, late severe generalized and prominent bulbar signs and crisis.

Inclusion criteria were all patients with a diagnosis of myasthenia gravis to be treated with an elective thymectomy. Exclusion criteria were all patients who developed myasthenia crisis or respiratory failure who needed plasmapheresis or immunoglobulin and received a thymectomy in the same admission. The primary end point of the study was post-operative intubation time, and secondary end points were 30-day mortality, length of hospital stay, post-operative complications, and complications related to plasmapheresis. The variables collected for each patient include age, gender, underlying disease, Osserman classification, history of myasthenic crisis before surgery, ocular symptoms, muscle power according to the Medical Research Council (MRC) Scale for muscle strength [7] (Grade 5: muscle contracts normally against full resistance; Grade 4: muscle strength is reduced, but muscle contraction can still move joint against resistance; Grade 3: muscle strength is further reduced such that the joint can be moved only against gravity with the examiner's resistance completely removed. As an example, the elbow can be moved from full extension to full flexion starting with the arm hanging down at the side; Grade 2: muscle can move only if the resistance of gravity is removed. As an example, the elbow can be fully flexed only if the arm is maintained in a horizontal plane; Grade 1: only a trace or flicker of movement is seen or felt in the muscle or fasciculations are observed in the muscle; Grade 0: no movement is observed), bulbar and respiratory involvement, medication (anticholinesterase, steroid, and immunosuppressant), functional vital capacity (FVC) within one week before surgery, type of operation, operative time, blood loss, post-operative intubation time, length of intensive care unit (ICU) stay, length of hospital stay, complications, pathology report, and 30-day mortality.

A thymectomy was performed by the single surgical team under standard general anesthesia for myasthenia patient protocols. There were four approaches for thymectomy including median sternotomy, collar incision, right-sided video-assisted thoracoscopic surgery (VATS), and right-sided thoracotomy. All parts of the thymus gland including both superior and inferior horns were removed with the surrounding fatty tissue and pericardial fat patch. For the median sternotomy and collar incision approaches, two phrenic nerves were identified, laterally for being the landmarks of dissection. For VATS and thoracotomy, only the right phrenic nerve was identified but the fat was removed until the left mediastinal pleura were clearly identified which meant no remaining left pericardial fat patch was seen. Postoperatively the tracheal extubation was considered in all cases depending on clinical and respiratory variables such as adequate respiration, tidal volume, and inspiratory force. All patients were transferred to sub-ICU or ICU and received the same dosage of preoperative pyridostigmine, prednisolone, or azathioprine after surgery. 

Statistical analysis was performed using the STATA version 11.0. The normally distributed continuous variables were expressed as a mean ± standard deviation and were analyzed by *t*-test. Nonnormally distributed continuous variables were expressed as medians and interquartile ratio (IQR) and were analyzed by rank sum test. Categorical variables were analyzed by Fisher's exact test. Logistic regression was used to calculate the propensity score, which evaluates confounding by indication. The propensity score was calculated by matching of the age, sex, Osserman classification, history of myasthenic crisis at any time and one month before surgery, grading of muscle weakness in any part, respiratory involvement, history of myasthenic crisis any time and within one month, history of plasmapheresis within one month, and FVC within one week before surgery. Multivariable exponential risk regression analysis weighted with a propensity score was used for comparing the primary end point between both groups. A *P* value less than 0.05 was considered to be significant. There are no missing data because we have standard record form to record all variables which were used in this study. 

## 3. Results

 Eighty-six myasthenia patients were enrolled in this study including 61 females (71.0%) and 25 males (29.0%). All patients were divided into two groups: 33 patients in the PPG and 53 patients in the NPPG. Demographic data were shown in Table 1. There were no statistically significant differences between the two groups in terms of gender, age, underlying disease, Osserman classification, ocular involvement, and use of preoperative medication. 

 Patients in the PPG group had a significantly greater history of myasthenic crisis at any time and within one month of surgery than patients in the NPPG (48.5% versus 22.6%, *P*-value of 0.018 and 21.2% versus 5.7%, *P*-value of 0.005, resp.). Furthermore, patients in the PPG group had a significantly greater history of plasmapheresis within one month before surgery than patients in the NPPG (33.3% versus 9.4%, *P*-value of 0.009). 

The grading of muscle weakness (neck, upper and lower extremities) in the PPG was significantly more than that in the NPPG. In the PPG, the number of patients who had respiratory involvement and bulbar involvement was significantly more than that in the NPPG (18.2% versus 1.9%, *P* = 0.012 and 39.4% versus 11.3%, *P* = 0.003, resp.). The FVC measured before surgery in the PPG was significantly lower than that in the NPPG (1503.3 ± 304.7 versus 2552.9 ± 221.5, *P* = 0.019). 

Propensity score (probability of receiving treatment) which was calculated by multivariable logistic regression analysis by matching of the age, sex, Osserman classification, history of myasthenic crisis at any time and one month before surgery, grading of muscle weakness in any part, respiratory involvement, history of myasthenic crisis any time and within one month, history of plasmapheresis within one month, and FVC within one week before surgery showed statistically significant differences (*P* value < 0.001) shown in Table 1. The meaning of differences in propensity score signifies that the overall characteristics of patients in each group were different (heterogeneous population). Consequently, we used a propensity score for weighting of all factors in the multivariable exponential risk regression analysis for the identification of the post-operative outcomes as shown in Table 3. 

Comparisons of the operative and the post-operative data are shown in Table 2. The type of operation was significantly different between both groups. The most common procedure in the PPG was a median sternotomy (54.6%) and in the NPPG was VATS thymectomy (58.5%). The choice of procedures depended on surgeon preference; however since 2008, we only perform VATS thymectomy. There were no differences in operative time and blood loss between the two groups. Most of patient were extubated immediately after the operation (87.9% in PPG and 94.3% in NPPG) without significant differences between both groups (*P* = 0.421). 

Certain life-threatening complications that have been reported, such as cardiac arrhythmia and asystole, respiratory arrest, seizure, anaphylactic and febrile reactions, hemorrhagic and thrombotic episodes, and fluid imbalance, did not occur in our series. There were three patients who had post-operative complications in our study, two in the PPG (one was a catheter-related complication, false aneurysm at the puncture site, and another patient was reintubated due to a myasthenic crisis and died due to pneumonia and septicemia), and one patient in the NPPG who had a postoperative respiratory infection, pneumonia, and septicemia and died on the 15th post-operative day. However, there was no significant difference in post-operative complications between the two groups. The mean of ICU stays was 2.3 days in the PPG and one day in the NPPG for observing postoperative complications (*P* = 0.384). The mean of hospital stays was 6.1 days in the PPG and 5.2 days in the NPPG (*P* = 0.187). 

The result of post-operative outcome calculated by multivariable exponential risk regression analysis weighting with propensity score is shown in Table 3. There were no significant differences between the two groups in terms of immediate post-operative extubation, complications, 30-day mortality, and length of hospital stay. 

## 4. Discussion 

 Thymectomy is widely used as a valuable treatment modality for generalized myasthenia gravis with long-term remission. Patients with myasthenic weakness require preoperative plasmapheresis to improve peri-operative outcomes (complications, duration of hospital stay and overall morbidity) [8–10]. Although plasmapheresis is a useful treatment modality, there are several disadvantages including the requirement of human protein replacement which increases the risk of anaphylaxis, transfusion-transmitted contagion such as hepatitis and HIV infection, hemolysis, catheter-related complication, hypotension, and high cost, and there is no consensus that plasmapheresis before thymectomy improves peri-operative outcomes [11].

In this retrospective study, patients in the PPG group had a significantly greater history of myasthenic crisis at any time and within one month than patients in NPPG, and patients in PPG group had a significantly greater history of plasmapheresis within one month before surgery than patients in NPPG. This result represented confounding by indication. Emergency thymectomy were not performed in some patients who had myasthenic crisis, approximately 32% in this study, because their clinical symptoms were rapidly improved after treating the precipitating cause, therefore, thymectomy were performed as elective cases. Also some patients' clinical picture improves significantly after plasmapheresis, and they preferred to be scheduled for an elective thymectomy. Furthermore, patients in the NPPG had a greater degree of muscle weakness than those in the PPG. Moreover, the number of patients in the PPG who had respiratory and bulbar involvement was greater than that in the NPPG. This is the reason why patients in the PGG had significantly increased plasmapheresis performed within one month before surgery, a history of myasthenic crisis at any time and within one month before surgery than in the NPPG.

 VATS thymectomy has been performed in our facility since 2008; therefore fifty percent of cases were VATS approach, and this may be the reason why there were statistically significant differences in this approach. 

The heterogeneous population was shown between both groups because there were multiple confounders by indication; therefore, we used propensity score matching of age, sex, Osserman classification, history of myasthenic crisis at any time and one month before surgery, grading of muscle weakness in any part, respiratory involvement, history of myasthenic crisis any time and within one month, history of plasmapheresis within one month, and FVC within one week before surgery and calculated by multivariable logistic regression analysis. The propensity score was used to weight all factors and was included in multivariable exponential risk regression analysis for the identification of post-operative outcomes including immediate extubation after surgery, post-operative complications, 30-day mortality, and length of hospital stay. The results showed no statistically significant differences in post-operative complications, 30-day mortality, length of hospital stay, and immediate extubation after surgery; however, patients in the PPG group had greater opportunities for immediate extubation after surgery than those in the NPPG (risk ratio (RR) 2.94, 95% confident interval (CI) of 0.98–8.79, *P* value of 0.054).

The frequency of overall complication rates in this study was 3.5% (3 in 86); there was only one complication in the NPPG (2%) and 6% in the PPG. In a previous study Vucic and Davis [12] report a different complication rate of 24.7% in 73 patients who underwent a thymectomy. El-Bawab et al. [10] studied the routine and selective use of plasmapheresis before thymectomy and reported that the complication rate in a routine use of the plasmapheresis group was 25% and the catheter-related complications occurred in 4% of the population which is comparable with 3% in our study. The complication for our study was a pseudoaneurysm of the femoral artery at the puncture site requiring open surgical repair. 

 Yeh et al. [8] and Nagayasu et al. [9] suggested that preoperative plasmapheresis may facilitate improved outcomes of patients with myasthenia gravis after thymectomy; however our study showed that the post-operative outcome was not different between the two groups. There was no difference in immediate post-operative extubation time, length of hospital stay, and 30-day mortality. This indicated that preoperative plasmapheresis for elective thymectomy in myasthenia gravis may not be necessary, even though the patients have a history of crisis. Our results were similar to El-Bawab et al. [10] that reported no difference in both groups in terms of overall median time of mechanical ventilation, ICU stay, and hospital stay. 

Limitations of this study include the retrospective nature of the study and selection bias of patients. The power to detect difference of propensity score between both groups is only 60 percent because of small sample size. Although we used a propensity score for weighting all patient characteristics in both groups, there may have been some unknown factors which could affect the result of this study.

In conclusion, elective thymectomy without preoperative plasmapheresis in myasthenia gravis patient regardless of a history of myasthenic crisis did not affect the overall outcomes when compared with preoperative plasmapheresis and may reduce catheter-related and post-operative complications. Therefore, preoperative plasmapheresis may be unnecessary in all elective thymectomies, even though patients have a history of crisis. However, patients who have history of myasthenic crisis more than one time or a significantly low motor power grading of extremities, or bulbar involvement, should be considered for preoperative plasmapheresis. The results of this study do not represent patients who have a myasthenic crisis before emergency or urgent surgery, repeated myasthenic crisis, or bulbar involvement. Prospective studies to include a larger number of patients are needed for definite conclusions.

## Figures and Tables

**Table 1:** Characteristics of patients between both groups.

Characteristics	Preoperative with plasmapheresis group (PPG) *N* (33)	Preoperative without plasmapheresis group (NPPG) *N* (53)	*P* value
Male, *n* (%)	8 (24.2)	17 (32.1)	0.475
Age (years), (mean ± SD)	44.2 ± 11.6	44.0 ± 15.3	0.933
Underlying disease (DM or HT or dyslipidemia)	17 (51.5)	30 (56.6)	0.663
Osserman classification			0.611
I	2 (6.1)	5 (9.4)	0.703
IIA	6 (18.2)	14 (26.4)	0.440
IIB	21 (63.6)	31 (58.5)	0.658
III	4 (12.1)	3 (5.7)	0.421
History of myasthenic crisis at any time	16 (48.5)	12 (22.6)	0.018
History of myasthenic crisis within one month before surgery	7 (21.2)	1 (1.9)	0.005
History of plasmapheresis within one month before surgery	11 (33.3)	5 (9.4)	0.009
Ocular involvement	15 (45.5)	25 (47.2)	1.000
Neck muscle motor power (mean ± SD)	4.5 ± 0.7	4.8 ± 0.4	0.025
Upper extremities motor power (mean ± SD)	4.4 ± 0.7	4.7 ± 0.5	0.009
lower extremities motor power (mean ± SD)	4.3 ± 0.8	4.7 ± 0.5	0.004
Respiratory involvement	6 (18.2)	1 (1.9)	0.012
Bulbar involvement	13 (39.4)	6 (11.3)	0.003
Mestinon use	33 (100)	47 (88.7)	0.078
Dose of mestinon (mg/day) (mean ± SD)	238.2 ± 132.0	204.9 ± 88.1	0.179
Prednisolone use	30 (91.0)	48 (91.0)	1.000
Dose of prednisolone (mg/day) (mean ± SD)	36.0 ± 19.8	29.3 ± 14.9	0.094
Azathioprine use	16 (48.5)	21 (39.6)	0.503
Dose of Azathioprine (mg/day) (mean ± SD)	65.6 ± 22.1	73.8 ± 28.0	0.342
FVC (mean ± SD)	1503.3 ± 304.7	2552.9 ± 221.5	0.019
Probability of receiving treatment (propensity score)*	0.53 ± 0.32	0.26 ± 0.15	<0.001

DM: diabetic mellitus, HT: hypertension, FVC: functional vital capacity.

*Propensity score calculated by logistic regression analysis considering gender, age, underlying disease, Osserman classification, history of myasthenic crisis at any time and within one month before surgery, ocular involvement, neck muscle, upper and lower extremities motor power, respiratory involvement, bulbar involvement, drug used, and FVC as determinants of receiving preoperative plasmapheresis.

**Table 2:** Comparison of operative and postoperative data between both groups.

Characteristics	PPG *N* (33)	NPPG *N* (53)	*P* value
Operation			
Median sternotomy	18 (54.6)	19 (35.9)	0.118
VATS	4 (12.1)	31 (58.5)	<0.001
Collar incision	9 (27.3)	1 (1.9)	0.001
Thoracotomy	2 (6.1)	2 (3.8)	0.636
Operative times (minutes)	101.5 ± 37.6	103.5 ± 26.7	0.781
Blood loss (mL)	127.3 ± 26.5	91.7 ± 10.5	0.154
Extubation in operating room	29 (87.9)	50 (94.3)	0.421
ICU stay (days)	2.3 ± 1.2	0.8 ± 0.2	0.384
Postoperative complication			0.131
No complication	31 (93.9)	52 (98.1)	0.524
Bleeding	1 (3.0)	0 (0)	0.384
Infection	0 (0)	1 (1.9)	1.000
Myasthenic crisis	1 (3.0)	0 (0)	1.000
Cholinergic crisis	0 (0)	0 (0)	—
Reintubation	1 (3.0)	1 (1.9)	1.000
Length of hospital stay (days)	6.1 ± 4.2	5.2 ± 2.3	0.187
Pathologic report			0.027
Thymoma	4 (12.1)	11 (20.8)	0.388
Thymic carcinoma	5 (15.2)	1 (1.9)	0.029
Thymic hyperplasia	7 (21.2)	4 (7.6)	0.096
Fatty involution	17 (51.5)	35 (66.0)	0.257
Others	0 (0)	2 (3.77)	0.521
30-day mortality	1 (3.0)	1 (1.9)	1.000

VATS: video-assisted thoracoscopic surgery.

**Table 3:** Postoperative outcomes of no plasmapheresis or immunoglobulin group compared with plasmapheresis or immunoglobulin group adjusted by propensity score.

Variables	Risk ratio	95% Confidence interval	*P* value
Immediate extubation after surgery (adjusted for PS^a^)	2.94	0.98–8.79	0.054
Postoperative complications (adjusted for PS^a^)	1.00	0.25–3.97	0.989
30-day mortality (adjusted for PS^a^)	0.05	0.00–13.63	0.296
Length of hospital stay* (adjusted for PS^a^)	0.64	(−0.63)–1.90	0.325

PS^a^: propensity score calculated by logistic regression analysis considering gender, age, underlying disease, Osserman classification, history of myasthenic crisis at any time and within one month before surgery, ocular involvement, neck muscle, upper and lower extremities motor power, respiratory involvement, bulbar involvement, drug used, and FVC as determinants of receiving preoperative plasmapheresis.

*Mean difference.
